# The Role of Monocytes and Macrophages in Human Atherosclerosis, Plaque Neoangiogenesis, and Atherothrombosis

**DOI:** 10.1155/2019/7434376

**Published:** 2019-04-04

**Authors:** Francesco Moroni, Enrico Ammirati, Giuseppe Danilo Norata, Marco Magnoni, Paolo G. Camici

**Affiliations:** ^1^Vita-Salute University and San Raffaele Hospital, Milan, Italy; ^2^De Gasperis Cardio Center and Transplant Center, Niguarda Hospital, Milan, Italy; ^3^Department of Excellence for Pharmacological and Biomolecular Sciences, Università degli Studi di Milano, Milan, Italy; ^4^SISA Center for the Study of Atherosclerosis, Bassini Hospital, Cinisello B, Italy

## Abstract

Atherosclerosis is one of the leading causes of death and disability worldwide. It is a complex disease characterized by lipid accumulation within the arterial wall, inflammation, local neoangiogenesis, and apoptosis. Innate immune effectors, in particular monocytes and macrophages, play a pivotal role in atherosclerosis initiation and progression. Although most of available evidence on the role of monocytes and macrophages in atherosclerosis is derived from animal studies, a growing body of evidence elucidating the role of these mononuclear cell subtypes in human atherosclerosis is currently accumulating. A novel pathogenic role of monocytes and macrophages in terms of atherosclerosis initiation and progression, in particular concerning the role of these cell subsets in neovascularization, has been discovered. The aim of the present article is to review currently available evidence on the role of monocytes and macrophages in human atherosclerosis and in relation to plaque characteristics, such as plaque neoangiogenesis, and patients' prognosis and their potential role as biomarkers.

## 1. Introduction

In spite of the great advances in terms of prevention, diagnosis, and treatment of cardiovascular diseases (CVD) obtained in the last decades, diseases affecting the heart and vessels continue to exact a high toll in terms of morbidity and mortality worldwide [1]. Among CVD, atherosclerosis-related conditions including acute coronary syndromes (ACS) or stroke currently dominate mortality and disability statistics [1]. Atherosclerosis is a chronic, degenerative disease of large- and medium-sized arteries. The initiation of the atherosclerotic process, i.e., atherogenesis, involves the deposition of low-density lipoprotein (LDL) cholesterol into the subendothelium. LDL deposition appears to be more likely in regions of turbulent flow and low shear stress, which, through incompletely understood mechanotransduction pathways, [2] activates endothelial cells towards a proatherogenic phenotype [3]. Several environmental factors including high blood pressure or smoking contribute to endothelial dysfunction and thus support atherogenesis. The discovery of the pivotal role of LDL in the development of atherosclerosis has led to the development and implementation of effective lipid-lowering strategies, which reduce CVD morbidity and mortality [4]. Strategies aimed at controlling other known cardiovascular risk factors such as hypertension or smoking have led to a decrease in CVD burden [5, 6]. More recently, the role of inflammation in the process of atherogenesis has gained increasing interest. Lipid accumulation into the arterial wall promotes inflammation that involves the local and systemic activation of innate and adaptive immune response [7]. Although controlling hypercholesterolemia by lipid-lowering therapies reduces inflammation, [8] the elucidation of the role of immunity in atherosclerosis has in recent times paved the way for the development of innovative and effective preventive strategies, the most outstanding example of which is the use of a pure anti-inflammatory drug, canakinumab, an anti-interleukin- (IL-) 1beta antibody, in the secondary prevention of ACS [9]. Although the body of knowledge regarding the relation between atherosclerosis and immunity in humans is growing rapidly, a relevant proportion of it is derived from studies carried out in animal models of CVD. The results obtained therein are, however, not fully transferrable to the human setting due to intrinsic biologic, genomic, and environmental differences. Therefore, a careful characterization of human pathological specimens together with a detailed profiling of peripheral blood subsets is critical to address the potential role of immune cells in human atherosclerosis. The aim of the present review is to discuss the evidence supporting the role of monocytes and macrophages as innate immune effectors in human atherosclerosis.

## 2. Macrophages Initiate Local Inflammation in Nascent Atherosclerotic Lesions

The human innate immune system is endowed with germline-encoded receptors to allow for the surveillance of exogenous pathogens or cell damage [10]. These receptors are expressed on the surface of immune cells patrolling the human organism, including macrophages and dendritic cells, and are known as pattern recognition receptors (PRR). They recognize pathogen-associated molecular patterns (PAMPs), such as lipopolysaccharide, a constituent of Gram-negative bacteria, or viral nucleic acids, or damage-associated molecular patterns (DAMPs), expressed by cells in response to noxious stimuli [11]. The inflammatory process initiated by the activation of PRR generally leads to the removal of the inciting stimulus, which however may occasionally persist and become maladaptive, leading to disease per se [7]. The activation of PRR in the context of the arterial wall is believed to be among the initiators of atherogenesis. The molecular patterns underlying the activation of the inflammatory response are only recently beginning to be elucidated. Infectious agents may contribute to provoking a response, but a primary role in atherogenesis is currently considered unlikely [12]. Other potential antigens, such as heat shock proteins (HSPs) and cholesterol crystals, have been implied, but as both require preexisting tissue damage, they likely contribute to amplify the inflammatory cascade, but not to the inflammation initiation [13, 14]. Currently available evidence points at epitopes generated by enzymatic and nonenzymatic oxidation of LDLs within the subendothelial space as major DAMPs involved in triggering the inflammatory cascade [15]. These oxidation-specific epitopes are recognized by a variety of PRR, among which Toll-like receptor 4 (TLR-4) deserves a special mention [15]. TLR-4 was in fact shown to be highly expressed in human atherosclerotic plaques, [16] and its expression was shown to be enhanced by LDLs [17]. Vice versa, the removal of cholesterol by HDLs has been shown to decrease TLR4 expression and macrophage activation [18]. On the other hand, however, the relation of HDLs and innate inflammation may not be straightforward. Indeed, HDLs were recently shown to exert a proinflammatory effect on mouse and human macrophages, possibly due to lipid raft disruption secondary to cholesterol depletion and subsequent activation of TLRs and protein kinase C signaling [19, 20]. Circulating monocytes of subjects with vulnerable atherosclerotic plaques in the coronary arteries express higher levels of TLR-4 when compared to subjects with stable coronary artery atherosclerosis [21]. Similarly, subjects with unstable angina had higher levels of expression of TLR-4 on circulating monocytes when compared to asymptomatic subjects with cardiovascular risk factors [22]. Interestingly, population genetics studies have initially suggested that the hypomorphic TLR-4 allele, leading to a blunted inflammatory activation, Arg299Gly was shown to be associated with the reduced risk of myocardial infarction [10]. However, a recent meta-analysis including 8299 patients suffering from acute myocardial infarction (AMI) and 6849 healthy controls failed to demonstrate any imbalance in the prevalence of TLR-4 Arg299Gly polymorphism among AMI patients [23].

## 3. Lipid-Laden Macrophages Contribute to the Development of Atherosclerotic Lesion

Blood monocytes and eventually resident vascular macrophages are the leukocytes that are recruited earlier in the nascent atherosclerotic lesion [24]. Direct evidence concerning the recruitment and activation of monocytes and macrophages in humans is currently unavailable, and most of our knowledge is derived from studies on animal models. Local activation of inflammation has been shown to induce the production of cytokines and chemokines, among which C-C motif chemokine ligand 2 (CCL2), also termed monocyte chemoattractant protein 1 (MCP-1), appears to play a major role [25–27]. These soluble chemotactic signals recruit circulating monocytes within the blood vessel wall through C-C chemokine receptor (CCR)2 and CCR4 [28]. Of note, studies performed in mouse models of atherosclerosis have shown that different subsets of monocytes are differentially recruited into the atherosclerotic plaque. Indeed, monocytes expressing high levels of surface lymphocyte antigen 6 complex, i.e., Ly6C^hi^ monocytes, appear to be the greatest contributors to plaque macrophages. The human orthologue of Ly6C^hi^ monocytes is the CD14^+^CD16^−^ monocytes, currently referred also as “classical” monocytes [29]. Their putative role appears to be proinflammatory [30] and was shown to be increased in specific dyslipidemic conditions [31]. While CD14^+^CD16^−^ monocytes have been initially reported to predict cardiovascular events, [32] a subsequent work showed that a different subset with more markedly inflammatory functions and with no murine counterpart, the intermediate CD14^hi^CD16^+^ monocytes, has been implicated as a key cell type in the development of atherosclerosis [30].

Classical histological studies have shown that monocytes and resident macrophages undergo local proliferation [33] and eventually mature and acquire a phagocytic phenotype. Lineage-tracing studies have indeed found that peripheral proliferation of macrophages is the dominant mechanism for macrophage increase in atherosclerotic lesions [34]. Once within the vessel wall, macrophages and monocyte-derived macrophages start scavenging oxidized LDL (OxLDL) [35]. The uptake of OxLDL is mediated by surface scavenging receptors, including scavenger receptor- (SR-) A1, SR-B2 (also termed CD36), and E1 (also termed lectin-like OxLDL receptor-1 (LOX-1)) [36]. Interestingly, these receptors seem to be under the transcriptional control of the nuclear factor- (NF-)-kB, an inflammatory master switch activated by both PRR and the effect of proinflammatory cytokines [37]. The uptake of cholesterol is at least in part counterbalanced by cholesterol efflux from the macrophages, which is mediated by the ATP-binding cassette (ABC) transporters A1 and G1. These transporters mediate the transfer of cholesterol to a free apolipoprotein A1 or directly to high-density lipoproteins containing either apoA1 or apolipoprotein E (high-density lipoprotein (HDL)) [35, 38]. HDLs mediate the transport of cholesterol towards the liver, a process known as reverse cholesterol transportation. This mechanism, which is peculiar for innate immune cells, is critical to control cellular cholesterol metabolism thus linking the activity of HDL and its components with the immune inflammatory response [39].

Indeed, an imbalance between cholesterol uptake and efflux leads to intracytoplasmic accumulation of cholesteryl lipid droplets [35]. This ultimately leads to the formation of lipid-laden foam cells, the hallmark of atherosclerosis [40]. The progression of cellular cholesterol loading leads to the triggering of an unfolded protein response in the endoplasmic reticulum, which brings cellular dysfunction. Cholesterol might precipitate within the cell as crystals and activate the inflammasome; this might lead to programmed cellular death, i.e., apoptosis or eventually necrosis [41]. Apoptosis and secondary necrosis lead to the development of an atherosclerotic necrotic core within the arterial lesion [42]. The necrotic core is mainly composed of cellular debris, [43] and lipid material [44] and therefore is highly thrombogenic. It is separated from the bloodstream by a fibrous cap. A discontinuation or rupture of the fibrous cap initiates a process of intraluminal thrombosis leading eventually to acute events including acute coronary syndromes or stroke. The rupture of the fibrous cap appears to be more likely when it is thinner or eventually infiltrated by foam cells [45].

Recent data suggested that lipid loading may begin even in circulating monocytes, which develop a foamy monocyte phenotype and subsequently migrate into the nascent atherosclerotic plaque. [46] Interestingly, the impairment of reverse cholesterol transportation, indirectly evaluated with serum cholesterol acceptor capacity, was shown to be associated to an increase in terms of cardiovascular death, nonfatal AMI, nonfatal stroke, or coronary revascularization at 9.4 years in a free living population of 2924 otherwise healthy subjects [47]. More recent data confirmed that serum cholesterol acceptor capacity, measured using cholesterol-loaded human THP-1 macrophages and patients' serum as a cellular cholesterol acceptor, is a strong independent determinant of cardiovascular morbidity and mortality. In a recent report on 1609 patients suffering from acute coronary syndrome, cholesterol efflux was significantly lower in subjects who died at 1.9 years of follow-up, despite comparable levels of HDL cholesterol. Indeed, serum cholesterol acceptor capacity was associated with in-hospital survival (hazard ratio (HR): 0.63, 95% confidence interval (CI): 0.40 to 0.97, *p* = 0.038) and lower 30-day mortality (HR: 0.32, 95% CI: 0.13 to 0.78, *p* = 0.012) after adjustment for cardiovascular risk factors [48].

## 4. Monocytes and Macrophages Contribute to Atherosclerotic Plaque Neoangiogenesis

The formation of a lipid-rich necrotic core within the arterial wall in the course of atherogenesis necessarily brings the formation of a hypoxic environment. The physiological response to hypoxia is a complex biological process leading to the formation of new blood vessels, i.e., neoangiogenesis [49]. Interestingly, intense neoangiogenesis takes place within the atherosclerotic plaque [50]. Of note, highly neovascularized plaques appear more prone to rupture and eventually give rise to acute atherothrombotic complications [51]. Indeed, intraplaque hemorrhage is a well-established process leading to the progression from stable atherosclerotic lesions to unstable, high-risk plaques [52]. Red blood cells within the plaque provide excess cholesterol and phospholipids within the plaque, causing the expansion of the necrotic core and fostering further activation of inflammation [52]. Inflammation per se has been shown to play a fundamental role in the process of neoangiogenesis, [53] and macrophages in particular have been shown to be pivotal in the formation of new blood vessels [54, 55]. Locally, innate immune cells secrete proangiogenic growth factors, such as vascular endothelial growth factor (VEGF) and basic fibroblast growth factor (bFGF) [56]. Furthermore, they secrete matrix metalloproteinases, which assist new vessel sprouting by degrading and remodeling the extracellular matrix and eventually activating or degrading growth factors [57, 58]. The resulting blood vessels appear inherently dysfunctional, thus allowing for blood leakage, fostering plaque expansion, new hypoxia, and further angiogenesis [51]. Indeed, the association between hypoxia, macrophage plaque infiltration, and neoangiogenesis has been demonstrated in human through histological studies [59]. Indeed, in a groundbreaking study by Sluimer et al., hypoxia was demonstrated in human carotid artery atherosclerotic plaques of 7 subjects undergoing carotid endarterectomy through the use of pimonidazole assay [59]. The detection of hypoxia strongly correlated with the presence of CD68-expressing macrophages and the presence of neoangiogenesis and of thrombus apposition on the plaque [59]. In addition, atherosclerotic plaque neovascularization can be directly visualized in vivo in large arteries in humans, i.e., the carotid arteries. This is made possible through the use of dedicated echographic techniques employing microbubble-based contrast media or using dedicated magnetic resonance imaging (MRI) protocols [60–62]. Microbubbles are strictly intravascular; therefore, the visualization of a contrast material within the plaque core implies the presence of neovessels. On the other hand, dedicated MRI protocols allow the detection of paramagnetic contrast media into the plaque, allowing the indirect visualization of the leaky neovessels [62]. To the best of our knowledge, only two studies have analyzed the relation between monocyte subpopulations in vivo and the identification of plaque neovessels in the carotid arteries using contrast-enhanced ultrasound (CEUS). In a study by Jaipersad and colleagues on 160 subjects, the CD14^+^CD16^−^CCR2^+^ subset was associated with a more severe plaque and more abundant neovascularization [63]. A subsequent study by our group on the other hand, including 55 patients with an intermediate carotid artery stenosis with overall 255 carotid lesions, showed that patients with evidence of a more intense carotid artery plaque neovascularization had lower overall levels of circulating monocytes, which was mainly due to a reduction of CD14^hi^CD16^−^ classical monocytes [64]. This pattern suggested a potential redistribution of inflammatory cells within highly active, neovascularized plaques. Indeed, in a subsequent proof of a principle study including 9 subjects with intermediate carotid artery plaques and no current indication to carotid revascularization, we were able to show that in subjects with atherosclerosis and reduced circulating CD14^hi^CD16^−^ monocytes, carotid plaques are indeed enriched with activated macrophages, which indeed supports our redistribution hypothesis [65]. A study on 32 subjects undergoing comprehensive carotid plaque evaluation using hybrid Positron Emission Tomography/Computed Tomography (PET/CT) imaging with ^15^F-fluorodeoxyglucose (FDG) and MRI showed a linear correlation in terms of FDG uptake, an imaging marker of inflammation, and neovascularization [62]. Interestingly, in the subset of patients undergoing carotid endarterectomy, the amount of neovascularization strongly correlated with plaque macrophage infiltration and plaque major histocompatibility complex (MHC) II, a marker of plaque inflammation [62].

## 5. Circulating Monocyte Subsets in Human Atherosclerosis

**Figure 1:**
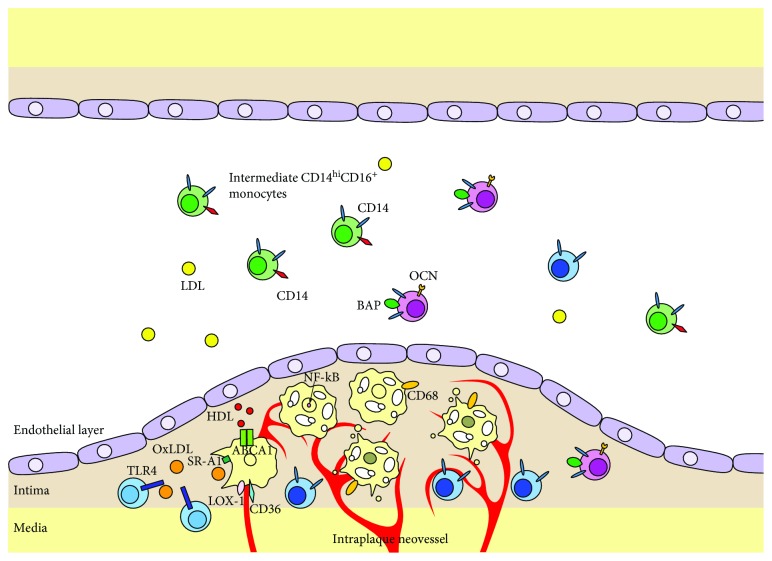
Summary of the main findings of human studies concerned with monocytes and macrophages and atherosclerosis. In subjects with high risk plaques, intermediate CD14^hi^CD16^+^ monocytes are enriched in the circulation. Less evidence is available for osteogenic biomarker expressing CD14^+^BAP^+^OCN^+^ monocytes, which appear to be enriched in the peripheral blood of subjects with large necrotic cores within the plaque. On the other hand, in subjects with highly neovascularized plaques, classical CD14^hi^CD16^−^ monocytes are reduced in the circulation, possibly due to the redistribution into the plaque. Monocytes are activated by OxLDL acting through pattern recognition receptors (PRR). The PRR most frequently implicated in human cardiovascular disease is TLR4. Monocytes locally differentiate into macrophages, which take up OxLDL by means of scavenging receptors. Cholesterol is then transported outside the cell through specialized transporters, including ABCA1. ABCA1 transports cholesterol to nascent HDLs. When cholesterol loading exceeds macrophage efflux capability, the macrophage turns into a foam cell. Human plaques are enriched with NF-kB-expressing macrophages.

Several human studies have tried to identify a polarization of a circulating monocyte subpopulation, mainly through the characterization of the expression of cell surface markers using flow cytometry. A high number of circulating monocytes were per se shown to be associated to a higher risk of cardiovascular events in subjects with known coronary artery disease [66]. A recent study by Justo-Junior and colleagues on 100 subjects showed that individuals with unstable angina had a higher number of circulating intermediate CD14^hi^CD16^+^ monocytes [22]. In addition, intermediate monocytes of these patients expressed higher surface concentration of chemokine receptors, including CCR2, and of PRR, in particular TLR-4 [22]. Again, a study by Zhuang et al. on 79 patients undergoing coronary angiography for acute coronary syndromes compared with 33 subjects with no evidence of coronary artery disease showed that the patients had higher circulating numbers of intermediate CD14^hi^CD16^+^ monocytes [67]. In addition, patients with a thin cap fibroatheroma were shown to have the largest number of circulating CD14^hi^CD16^+^ monocytes [67]. In a recent study by Ozaki et al. on 65 subjects undergoing coronary multidetector computed tomography, the proportion of circulating CD14^hi^CD16^+^ monocytes expressing TLR-4 was shown to be higher in subjects with plaque features of vulnerability [21]. However, a prospective study involving 191 subjects with chronic kidney disease was unable to demonstrate any association between the number of CD14^+^TLR-4^+^ monocytes and incident cardiovascular events [68]. A large study comprising 1546 asymptomatic subjects taking part in the Atherosclerosis Risk in Community Carotid Magnetic Resonance Imaging study found that circulating monocytes from patients with larger plaques expressed higher levels of TLR-2, while monocytes from patients with smaller plaques expressed higher quantities of CD14, TLR-4, and myeloperoxidase [69]. This observation might represent an indirect evidence that different cell types are involved at various stages of the atherosclerotic process [69]. A recent study assessed whether monocytes expressing osteogenic markers could also be associated with atherosclerosis [70]. Indeed, myeloid cells expressing osteogenic markers have been shown to contribute to calcium deposition in peripheral tissues and eventually the development of vascular calcifications [71]. Interestingly, the number of osteocalcin and bone alkaline phosphatase expressing monocytes was higher in subjects with plaque features of vulnerability on virtual histology. Moreover, an enrichment of these cells in the coronary blood was found in subjects in which a large necrotic core could be demonstrated [70]. In a recent study involving 175 subjects undergoing carotid endarterectomy for asymptomatic, severe carotid stenosis, however, the total count of circulating monocytes was not found to correlate with plaque features of vulnerability, including thin cap atheroma, large necrotic core, intraplaque hemorrhage, or high neovessel density [72]. The lack of association with plaque vulnerability features was also confirmed when monocyte subpopulations, based on CD14 and CD16 surface expression, were evaluated. Interestingly, monocyte subpopulations were not associated to the occurrence of major adverse cardiovascular events after at 3 years of follow-up [72].  Table 1 summarizes the above-mentioned studies.  Figure 1 provides a graphical overview of the available evidence.

## 6. Plaque Macrophages in Atherosclerosis

Macrophages exert an essential role in terms of phagocytic killing of pathogens and antigen presentation, therefore triggering an adaptive immune response. However, they also exert a primary tissue homeostasis function, including removal of cellular debris and adaptive remodeling of extracellular matrix [73]. Indeed, macrophages respond to environmental stimuli to acquire a proinflammatory or a homeostatic phenotype [73]. On this basis, macrophages have been traditionally subdivided into the M1 inflammatory subset and M2 protissue subset [74]. While this distinction fails to adequately comprise the entire macrophage biological complexity, it provides a general scheme to classify macrophage function. Early histological studies hinted at a strong activation of inflammatory pathways of macrophages within human atherosclerotic plaques, pointing at a M1 polarization [75]. On the other hand, more recent studies suggest lower levels of M2 macrophages within vulnerable plaques [76]. Interestingly, M1 macrophages were shown to be enriched in the areas pf plaque more prone to rupture, while M2 on the stable adventitial side of the plaque [77]. Indeed, also, the anatomical site of the plaque appears to influence the M1/M2 proportion, with carotid artery plaque containing a larger percentage of M1 macrophages with respect to femoral artery plaques [78]. On the other hand, a recent study analyzing 110 human aortic plaques showed that both M1 and M2 subtypes are associated to progressive atherosclerosis and vulnerable plaques, which underlines the fact that the dichotomic M1/M2 distinction may be over simplistic [79].

Few clinical data are available on the impact of plaque macrophage infiltration on cardiovascular outcome. They are mainly derived from carotid artery samples, due to the widespread indication of carotid endarterectomy for primary or secondary stroke prevention. The level of macrophage infiltration within the carotid plaque of 1640 patients undergoing carotid endarterectomy for secondary stroke prevention was shown to directly correlate with the clinical stroke risk profile [80]. On the other hand, patients with a more marked macrophage infiltration within a carotid artery plaque were shown to have a lower risk of 1-year restenosis after carotid endarterectomy in a prospective cohort including 500 subjects [81]. However, macrophage infiltration within atherosclerotic plaques was not shown to predict cardiovascular outcomes after 2.3 years of follow-up in a cohort of 818 subjects [82]. Interestingly, in the same cohort, plaque neovascularization and intraplaque hemorrhage were the strongest plaque-associated predictors of future cardiovascular events [82]. A more recent cohort of 286 patients undergoing carotid endarterectomy followed up for 3 years showed that the proportion of MMP12^+^ macrophages, not of overall macrophage infiltration, could predict the incidence of future adverse cardiovascular events [83]. Of note, the current advanced vascular imaging approaches allow for direct in vivo visualization of intraplaque macrophages [60]. In particular, hybrid imaging with computed tomography and positron emission tomography (CT-PET) using the macrophage-specific ligand PK11195 allowed the demonstration in vivo that recently symptomatic carotid artery plaques are more infiltrated by macrophages when compared to asymptomatic carotid artery plaques [84]. Given that it is now established that the simple distinction between M1 and M2 does not recapitulate the “real” functional status of macrophages in the atherosclerotic plaque, the investigation of specific macrophage signatures, by single cell analysis, will represent the next step to characterize macrophage function.

Table 2 summarizes the evidence on macrophages and human plaques.  Table 3 schematizes the available evidence concerned with monocytes and macrophages in human atherosclerosis.  Figure 1 provides a graphical overview of the available evidence.

## 7. Conclusions

Monocytes and macrophages have a pivotal role in atherosclerosis initiation and development. While the majority of currently available data are currently derived from animal studies, a growing body of evidence is elucidating the role of monocytes and macrophages in human CVD. While both cell types may be amenable for targeted treatment to abate cardiovascular disease in the future, the current data also support the use of monocyte and macrophage subpopulations as markers for an increased cardiovascular risk.

## Figures and Tables

**Table 1:** Summary of currently available studies on human circulating monocytes subsets and atherosclerosis.

Authors	Year	Sample size	Monocyte subpopulation of interest	Main result	Reference
Jaipersad et al.	2014	160 individuals with carotid artery stenosis	CCR2-expressing monocytes, subdivided into classical, intermediate, and nonclassical subsets	Classical CD14^hi^CD16^−^CCR2^+^ monocytes are associated with the degree of carotid stenosis and plaque neovascularization evidenced by CEUS	[63]
Ammirati et al.	2016	64 individuals with intermediate carotid artery stenosis	CD14^+^ monocytes subdivided into classical, intermediate, and nonclassical subsets	Total CD14^+^ monocytes and nonclassical CD14^hi^CD16^−^ monocytes are reduced in subjects with higher CEUS-evidenced neovascularization	[64]
Ammirati et al.	2018	9 individuals with intermediate carotid artery stenosis	CD14^+^ monocytes subdivided into classical, intermediate, and nonclassical subsets	People with a stronger uptake of activated macrophage-specific radiotracer PK11195 had reduced circulating total CD14^+^ monocytes and nonclassical CD14^hi^CD16^−^ monocytes	[65]
Justo-Junior et al.	2018	18 controls, 34 individuals with CVRF, 32 patients with stable angina, and 16 patients with unstable angina	Classical, intermediate, and nonclassical circulating monocytes	Intermediate CD14^hi^CD16^+^ monocytes were elevated in unstable angina patients. They also displayed high expression of PRRs	[22]
Yamamoto et al.	2016	389 CAD patients	Peripheral blood monocytes	A high monocyte count predicted future cardiovascular events in CAD patients	[66]
Zhuang et al.	2017	48 patients with unstable angina and 31 patients with STEMI	Classical, intermediate, and nonclassical monocytes	Patients with evidence of thin cap fibroatheroma on OCT had significantly mode circulating intermediate CD14^hi^CD16^+^ monocytes	[67]
Lorenzen et al.	2011	191 subjects undergoing hemodialysis	CD14^+^TLR4^+^ monocytes	No association between CD14^+^TLR4^+^ and cardiovascular deaths or cardiovascular events	[68]
Collin et al.	2015	23 patients with nonobstructive CAD undergoing IVUS	CD14^+^BAP^+^OCN^+^ monocytes	CD14^+^BAP^+^OCN^+^ monocytes correlate with the presence of a large necrotic core within coronary artery plaques	[70]
Meeuwsen et al.	2019	175 subjects undergoing carotid endarterectomy (85% symptomatic, 15% asymptomatic)	Total, classical, intermediate, and nonclassical monocytes	No association between monocyte subpopulations and plaque features of vulnerability. No association between monocyte subpopulations and major adverse cardiovascular events at 3 years of follow-up	[72]

CEUS: contrast-enhanced ultrasound; CAD: coronary artery disease; CVRF: cardiovascular risk factors; OCT: optical coherence tomography; IVUS: intravascular ultrasound.

**Table 2:** Summary of currently available clinical studies on human plaque macrophages.

Authors	Year	Sample size	Main finding	Reference
Brand et al.	1996	18 subjects	Atherosclerotic lesion in the aorta and carotid arteries contains a large proportion of NF-kB-positive macrophages (i.e., M1 macrophages) when compared to healthy arterial segments	[75]
Johnson et al.	2014	79 CEA specimens	Vulnerable lesions with a large necrotic core contained less MMP14^lo^TIMP3^hi^-M2 macrophages	[76]
Stöger et al.	2012	22 ruptured carotid atherosclerosis specimens and 22 adjacent stable controls	In carotid plaques, CD68+HLA-DP/Q/R+ M1 macrophages are enriched near the rupture-prone shoulder. On the other hand, M2 macrophages were enriched on the adventitial side of the vessel	[77]
Shaikh et al.	2012	32 carotid endarterectomy specimens, 25 femoral artery endarterectomy specimens	Carotid artery plaques have a larger necrotic core and contain more M1 macrophages when compared to femoral artery plaques	[78]
van Dijk et al.	2016	110 human perirenal aortic plaques	M2 and M1 macrophages are both enriched within progressive and vulnerable atherosclerotic plaques	[79]
Howard et al.	2015	1640 carotid artery plaques from patients undergoing CEA for secondary stroke prevention	CD68+ macrophage plaque content was associated with a 5-year stroke risk based on the ECST patient database	[80]
Hellings et al.	2008	500 carotid endarterectomy specimens	A high macrophage infiltration was associated with a high risk of ipsilateral carotid restenosis	[81]
Hellings et al.	2010	818 carotid endarterectomy specimens	Macrophage infiltration did not predict the 3-year risk of cardiovascular death or nonfatal stroke or nonfatal myocardial infarction. On the other hand, plaque neovascularization was associated with the relevant outcomes	[82]
Scholtes et al.	2012	236 subjects undergoing carotid endarterectomy for secondary stroke prevention	The proportion of MMP12+ macrophages was associated with major adverse cardiovascular events and stroke at 3 years	[83]
Gaemperli et al.	2012	9 patients with stroke due to carotid atherosclerosis and 27 subjects with asymptomatic carotid atherosclerosis	In vitro imaging of plaque macrophage infiltration using the macrophage-specific tracer PK11195 allowed to discriminate symptomatic vs. asymptomatic subjects	[84]

M1: proinflammatory M1 macrophages; M2: homeostasis-promoting M2 macrophages; CEA: carotid endarterectomy; MMP: matrix metalloproteinase; TIMP: tissue inhibitor of matrix metalloproteinase.

**Table 3:** Summary of the main findings on the relation between monocytes/macrophages and human atherosclerosis.

Monocyte/macrophage population	Cardiovascular events	Imaging features of vulnerability	Histologic features of vulnerability	Neovascularization
Total monocytes	+	NA	NA	+
Circulating CD14^hi^CD16^−^	NA	NA	NA	++
Circulating CD14^hi^CD16^+^	+	+	NA	NA
TLR4^+^ monocytes	—	NA	NA	NA
M1 macrophages	++	+	+++	NA
M2 macrophages	NA	NA	++	NA

A plus (+) represents a strong clinical evidence in favor of the association, while a minus (-) represents the failure of a well-designed study to establish an association. NA: not available.

## References

[1] GBD 2016 DALYs and HALE Collaborators (2017). Global, regional, and national disability-adjusted life-years (DALYs) for 333 diseases and injuries and healthy life expectancy (HALE) for 195 countries and territories, 1990-2016: a systematic analysis for the Global Burden of Disease Study 2016. *Lancet*.

[2] Chatzizisis Y. S., Coskun A. U., Jonas M., Edelman E. R., Feldman C. L., Stone P. H. (2007). Role of endothelial shear stress in the natural history of coronary atherosclerosis and vascular remodeling: molecular, cellular, and vascular behavior. *Journal of the American College of Cardiology*.

[3] Dai G., Kaazempur-Mofrad M. R., Natarajan S. (2004). Distinct endothelial phenotypes evoked by arterial waveforms derived from atherosclerosis-susceptible and -resistant regions of human vasculature. *Proceedings of the National Academy of Sciences of the United States of America*.

[4] Cholesterol Treatment Trialists' (CTT) Collaborators, Mihaylova B., Emberson J. (2012). The effects of lowering LDL cholesterol with statin therapy in people at low risk of vascular disease: meta-analysis of individual data from 27 randomised trials. *Lancet*.

[5] Turnbull F., Blood Pressure Lowering Treatment Trialists' Collaboration (2003). Effects of different blood-pressure-lowering regimens on major cardiovascular events: results of prospectively-designed overviews of randomised trials. *Lancet*.

[6] Pell J. P., Haw S., Cobbe S. (2008). Smoke-free legislation and hospitalizations for acute coronary syndrome. *New England Journal of Medicine*.

[7] Ammirati E., Moroni F., Magnoni M., Camici P. G. (2015). The role of T and B cells in human atherosclerosis and atherothrombosis. *Clinical & Experimental Immunology*.

[8] Catapano A. L., Pirillo A., Norata G. D. (2017). Vascular inflammation and low-density lipoproteins: is cholesterol the link? A lesson from the clinical trials. *British Journal of Pharmacology*.

[9] Ridker P. M., Everett B. M., Thuren T. (2017). Antiinflammatory therapy with canakinumab for atherosclerotic disease. *The New England Journal of Medicine*.

[10] Frantz S., Ertl G., Bauersachs J. (2007). Mechanisms of disease: Toll-like receptors in cardiovascular disease. *Nature Clinical Practice. Cardiovascular Medicine*.

[11] Falck-Hansen M., Kassiteridi C., Monaco C. (2013). Toll-like receptors in atherosclerosis. *International Journal of Molecular Sciences*.

[12] Song Z., Brassard P., Brophy J. M. (2008). A meta-analysis of antibiotic use for the secondary prevention of cardiovascular diseases. *The Canadian Journal of Cardiology*.

[13] Grundtman C., Kreutmayer S. B., Almanzar G., Wick M. C., Wick G. (2011). Heat shock protein 60 and immune inflammatory responses in atherosclerosis. *Arteriosclerosis, Thrombosis, and Vascular Biology*.

[14] Duewell P., Kono H., Rayner K. J. (2010). NLRP3 inflammasomes are required for atherogenesis and activated by cholesterol crystals. *Nature*.

[15] Miller Y. I., Choi S. H., Wiesner P. (2011). Oxidation-specific epitopes are danger-associated molecular patterns recognized by pattern recognition receptors of innate immunity. *Circulation Research*.

[16] Edfeldt K., Swedenborg J., Hansson G. K., Yan Z.-q. (2002). Expression of toll-like receptors in human atherosclerotic lesions: a possible pathway for plaque activation. *Circulation*.

[17] Doherty T. M., Fisher E. A., Arditi M. (2006). TLR signaling and trapped vascular dendritic cells in the development of atherosclerosis. *Trends in Immunology*.

[18] Catapano A. L., Pirillo A., Bonacina F., Norata G. D. (2014). HDL in innate and adaptive immunity. *Cardiovascular Research*.

[19] Smoak K. A., Aloor J. J., Madenspacher J. (2010). Myeloid differentiation primary response protein 88 couples reverse cholesterol transport to inflammation. *Cell Metabolism*.

[20] van der Vorst E. P. C., Theodorou K., Wu Y. (2017). High-density lipoproteins exert pro-inflammatory effects on macrophages via passive cholesterol depletion and PKC-NF-*κ*B/STAT1-IRF1 signaling. *Cell Metabolism*.

[21] Ozaki Y., Imanishi T., Hosokawa S. (2017). Association of Toll-like receptor 4 on human monocyte subsets and vulnerability characteristics of coronary plaque as assessed by 64-slice multidetector computed tomography. *Circulation Journal*.

[22] Justo-Junior A. S., Villarejos L. M., Lima X. T. V. (2019). Monocytes of patients with unstable angina express high levels of chemokine and pattern-recognition receptors. *Cytokine*.

[23] Yin Y. W., Sun Q. Q., Hu A. M., Liu H. L., Wang Q., Zhang B. B. (2014). Toll-like receptor 4 gene Asp299Gly polymorphism in myocardial infarction: a meta-analysis of 15,148 subjects. *Human Immunology*.

[24] Camici P. G., Rimoldi O. E., Gaemperli O., Libby P. (2012). Non-invasive anatomic and functional imaging of vascular inflammation and unstable plaque. *European Heart Journal*.

[25] Combadière C., Potteaux S., Rodero M. (2008). Combined inhibition of CCL2, CX3CR1, and CCR5 abrogates Ly6C^hi^ and Ly6C^lo^ monocytosis and almost abolishes atherosclerosis in hypercholesterolemic mice. *Circulation*.

[26] Takeya M., Yoshimura T., Leonard E. J., Takahashi K. (1993). Detection of monocyte chemoattractant protein-1 in human atherosclerotic lesions by an anti-monocyte chemoattractant protein-1 monoclonal antibody. *Human Pathology*.

[27] Yla-Herttuala S., Lipton B. A., Rosenfeld M. E. (1991). Expression of monocyte chemoattractant protein 1 in macrophage-rich areas of human and rabbit atherosclerotic lesions. *Proceedings of the National Academy of Sciences of the United States of America*.

[28] Tacke F., Alvarez D., Kaplan T. J. (2007). Monocyte subsets differentially employ CCR2, CCR5, and CX3CR1 to accumulate within atherosclerotic plaques. *The Journal of Clinical Investigation*.

[29] Ziegler-Heitbrock L., Ancuta P., Crowe S. (2010). Nomenclature of monocytes and dendritic cells in blood. *Blood*.

[30] Rogacev K. S., Cremers B., Zawada A. M. (2012). CD14++CD16+ monocytes independently predict cardiovascular events: a cohort study of 951 patients referred for elective coronary angiography. *Journal of the American College of Cardiology*.

[31] Sala F., Cutuli L., Grigore L. (2013). Prevalence of classical CD14++/CD16- but not of intermediate CD14++/CD16+ monocytes in hypoalphalipoproteinemia. *International Journal of Cardiology*.

[32] Berg K. E., Ljungcrantz I., Andersson L. (2012). Elevated CD14++CD16- monocytes predict cardiovascular events. *Circulation. Cardiovascular Genetics*.

[33] Gordon D., Reidy M. A., Benditt E. P., Schwartz S. M. (1990). Cell proliferation in human coronary arteries. *Proceedings of the National Academy of Sciences of the United States of America*.

[34] Robbins C. S., Hilgendorf I., Weber G. F. (2013). Local proliferation dominates lesional macrophage accumulation in atherosclerosis. *Nature Medicine*.

[35] Tabas I., Bornfeldt K. E. (2016). Macrophage phenotype and function in different stages of atherosclerosis. *Circulation Research*.

[36] Chistiakov D. A., Bobryshev Y. V., Orekhov A. N. (2016). Macrophage-mediated cholesterol handling in atherosclerosis. *Journal of Cellular and Molecular Medicine*.

[37] Hashizume M., Mihara M. (2012). Blockade of IL-6 and TNF-*α* inhibited oxLDL-induced production of MCP-1 via scavenger receptor induction. *European Journal of Pharmacology*.

[38] Norata G. D., Pirillo A., Catapano A. L. (2011). HDLs, immunity, and atherosclerosis. *Current Opinion in Lipidology*.

[39] Bonacina F., Coe D., Wang G. (2018). Myeloid apolipoprotein E controls dendritic cell antigen presentation and T cell activation. *Nature Communications*.

[40] Glass C. K., Witztum J. L. (2001). Atherosclerosis. the road ahead. *Cell*.

[41] Feng B., Yao P. M., Li Y. (2003). The endoplasmic reticulum is the site of cholesterol-induced cytotoxicity in macrophages. *Nature Cell Biology*.

[42] Clarke M. C. H., Bennett M. R. (2009). Cause or consequence: what does macrophage apoptosis do in atherosclerosis?. *Arteriosclerosis, Thrombosis, and Vascular Biology*.

[43] Gautier E. L., Huby T., Witztum J. L. (2009). Macrophage apoptosis exerts divergent effects on atherogenesis as a function of lesion stage. *Circulation*.

[44] Haka A. S., Grosheva I., Singh R. K., Maxfield F. R. (2013). Plasmin promotes foam cell formation by increasing macrophage catabolism of aggregated low-density lipoprotein. *Arteriosclerosis, Thrombosis, and Vascular Biology*.

[45] Bentzon J. F., Otsuka F., Virmani R., Falk E. (2014). Mechanisms of plaque formation and rupture. *Circulation Research*.

[46] Xu L., Dai Perrard X., Perrard J. L. (2015). Foamy monocytes form early and contribute to nascent atherosclerosis in mice with hypercholesterolemia. *Arteriosclerosis, Thrombosis, and Vascular Biology*.

[47] Rohatgi A., Khera A., Berry J. D. (2014). HDL cholesterol efflux capacity and incident cardiovascular events. *The New England Journal of Medicine*.

[48] Guerin M., Silvain J., Gall J. (2018). Association of serum cholesterol efflux capacity with mortality in patients with ST-segment elevation myocardial infarction. *Journal of the American College of Cardiology*.

[49] Bjornheden T., Levin M., Evaldsson M., Wiklund O. (1999). Evidence of hypoxic areas within the arterial wall in vivo. *Arteriosclerosis, Thrombosis, and Vascular Biology*.

[50] Kwon H. M., Sangiorgi G., Ritman E. L. (1998). Enhanced coronary vasa vasorum neovascularization in experimental hypercholesterolemia. *The Journal of Clinical Investigation*.

[51] Virmani R., Kolodgie F. D., Burke A. P. (2005). Atherosclerotic plaque progression and vulnerability to rupture: angiogenesis as a source of intraplaque hemorrhage. *Arteriosclerosis, Thrombosis, and Vascular Biology*.

[52] Kolodgie F. D., Gold H. K., Burke A. P. (2003). Intraplaque hemorrhage and progression of coronary atheroma. *The New England Journal of Medicine*.

[53] Silvestre J. S., Mallat Z., Tedgui A., Levy B. I. (2008). Post-ischaemic neovascularization and inflammation. *Cardiovascular Research*.

[54] Li A. C., Glass C. K. (2002). The macrophage foam cell as a target for therapeutic intervention. *Nature Medicine*.

[55] De Palma M., Naldini L. (2011). Angiopoietin-2 TIEs up macrophages in tumor angiogenesis. *Clinical Cancer Research*.

[56] Sunderkotter C., Goebeler M., Schulze-Osthoff K., Bhardwaj R., Sorg C. (1991). Macrophage-derived angiogenesis factors. *Pharmacology & Therapeutics*.

[57] Andreuzzi E., Colladel R., Pellicani R. (2017). The angiostatic molecule Multimerin 2 is processed by MMP-9 to allow sprouting angiogenesis. *Matrix Biology*.

[58] Bergers G., Brekken R., McMahon G. (2000). Matrix metalloproteinase-9 triggers the angiogenic switch during carcinogenesis. *Nature Cell Biology*.

[59] Sluimer J. C., Gasc J. M., van Wanroij J. L. (2008). Hypoxia, hypoxia-inducible transcription factor, and macrophages in human atherosclerotic plaques are correlated with intraplaque angiogenesis. *Journal of the American College of Cardiology*.

[60] Ammirati E., Moroni F., Pedrotti P. (2014). Non-invasive imaging of vascular inflammation. *Frontiers in Immunology*.

[61] Magnoni M., Ammirati E., Camici P. G. (2015). Non-invasive molecular imaging of vulnerable atherosclerotic plaques. *Journal of Cardiology*.

[62] Taqueti V. R., di Carli M. F., Jerosch-Herold M. (2014). Increased microvascularization and vessel permeability associate with active inflammation in human atheromata. *Circulation: Cardiovascular Imaging*.

[63] Jaipersad A. S., Shantsila A., Lip G. Y. H., Shantsila E. (2014). Expression of monocyte subsets and angiogenic markers in relation to carotid plaque neovascularization in patients with pre-existing coronary artery disease and carotid stenosis. *Annals of Medicine*.

[64] Ammirati E., Moroni F., Magnoni M. (2016). Circulating CD14+ and CD14^high^CD16− classical monocytes are reduced in patients with signs of plaque neovascularization in the carotid artery. *Atherosclerosis*.

[65] Ammirati E., Moroni F., Magnoni M. (2018). Carotid artery plaque uptake of 11C-PK11195 inversely correlates with circulating monocytes and classical CD14++CD16− monocytes expressing HLA-DR. *IJC Heart & Vasculature*.

[66] Yamamoto E., Sugiyama S., Hirata Y. (2016). Prognostic significance of circulating leukocyte subtype counts in patients with coronary artery disease. *Atherosclerosis*.

[67] Zhuang J., Han Y., Xu D. (2017). Comparison of circulating dendritic cell and monocyte subsets at different stages of atherosclerosis: insights from optical coherence tomography. *BMC Cardiovascular Disorders*.

[68] Lorenzen J. M., David S., Richter A. (2011). TLR-4+ peripheral blood monocytes and cardiovascular events in patients with chronic kidney disease--a prospective follow-up study. *Nephrology, Dialysis, Transplantation*.

[69] Matijevic N., Wu K. K., Howard A. G. (2011). Association of blood monocyte and platelet markers with carotid artery characteristics: the atherosclerosis risk in communities carotid MRI study. *Cerebrovascular Diseases*.

[70] Collin J., Gössl M., Matsuo Y. (2015). Osteogenic monocytes within the coronary circulation and their association with plaque vulnerability in patients with early atherosclerosis. *International Journal of Cardiology*.

[71] Fadini G. P., Albiero M., Menegazzo L. (2011). Widespread increase in myeloid calcifying cells contributes to ectopic vascular calcification in type 2 diabetes. *Circulation Research*.

[72] Meeuwsen J. A. L., de Vries J. J., van Duijvenvoorde A. (2019). Circulating CD14^+^CD16^−^ classical monocytes do not associate with a vulnerable plaque phenotype, and do not predict secondary events in severe atherosclerotic patients. *Journal of Molecular and Cellular Cardiology*.

[73] Mosser D. M., Edwards J. P. (2008). Exploring the full spectrum of macrophage activation. *Nature Reviews. Immunology*.

[74] Gordon S. (2003). Alternative activation of macrophages. *Nature Reviews Immunology*.

[75] Brand K., Page S., Rogler G. (1996). Activated transcription factor nuclear factor-kappa B is present in the atherosclerotic lesion. *The Journal of Clinical Investigation*.

[76] Johnson J. L., Jenkins N. P., Huang W.-C. (2014). Relationship of MMP-14 and TIMP-3 expression with macrophage activation and human atherosclerotic plaque vulnerability. *Mediators of Inflammation*.

[77] Stöger J. L., Gijbels M. J. J., van der Velden S. (2012). Distribution of macrophage polarization markers in human atherosclerosis. *Atherosclerosis*.

[78] Shaikh S., Brittenden J., Lahiri R., Brown P. A. J., Thies F., Wilson H. M. (2012). Macrophage subtypes in symptomatic carotid artery and femoral artery plaques. *European Journal of Vascular and Endovascular Surgery*.

[79] van Dijk R. A., Rijs K., Wezel A. (2016). Systematic evaluation of the cellular innate immune response during the process of human atherosclerosis. *Journal of the American Heart Association*.

[80] Howard D. P. J., van Lammeren G. W., Rothwell P. M. (2015). Symptomatic carotid atherosclerotic disease: correlations between plaque composition and ipsilateral stroke risk. *Stroke*.

[81] Hellings W. E., Moll F. L., De Vries J. P. (2008). Atherosclerotic plaque composition and occurrence of restenosis after carotid endarterectomy. *Journal of the American Medical Association*.

[82] Hellings W. E., Peeters W., Moll F. L. (2010). Composition of carotid atherosclerotic plaque is associated with cardiovascular outcome: a prognostic study. *Circulation*.

[83] Scholtes V. P. W., Johnson J. L., Jenkins N. (2012). Carotid atherosclerotic plaque matrix metalloproteinase-12-positive macrophage subpopulation predicts adverse outcome after endarterectomy. *Journal of the American Heart Association*.

[84] Gaemperli O., Shalhoub J., Owen D. R. J. (2012). Imaging intraplaque inflammation in carotid atherosclerosis with 11C-PK11195 positron emission tomography/computed tomography. *European Heart Journal*.

